# BV-GAN: 3D time-of-flight magnetic resonance angiography cerebrovascular vessel segmentation using adversarial CNNs

**DOI:** 10.1117/1.JMI.9.4.044503

**Published:** 2022-08-31

**Authors:** Dor Amran, Moran Artzi, Orna Aizenstein, Dafna Ben Bashat, Amit H. Bermano

**Affiliations:** a Tel-Aviv University, School of Electrical Engineering, Tel-Aviv, Israel; b Tel Aviv Sourasky Medical Center, Sagol Brain Institute, Tel Aviv, Israel; c Tel-Aviv University, Sackler Faculty of Medicine, Tel-Aviv, Israel; d Tel-Aviv University, Sagol School of Neuroscience, Tel-Aviv, Israel; e Tel Aviv Sourasky Medical Center, Neuroradiology Unit, Imaging Department, Tel Aviv, Israel; f Tel-Aviv University, Blavatnik School of Computer Science, Tel-Aviv, Israel

**Keywords:** anatomical attention, deep learning, segmentation, time-of-flight MR angiography, vessel

## Abstract

**Purpose:**

Cerebrovascular vessel segmentation is a key step in the detection of vessel pathology. Brain time-of-flight magnetic resonance angiography (TOF-MRA) is a main method used clinically for imaging of blood vessels using magnetic resonance imaging. This method is primarily used to detect narrowing, blockage of the arteries, and aneurysms. Despite its importance, TOF-MRA interpretation relies mostly on visual, subjective assessment performed by a neuroradiologist and is mostly based on maximum intensity projections reconstruction of the three-dimensional (3D) scan, thus reducing the acquired spatial resolution. Works tackling the central problem of automatically segmenting brain blood vessels typically suffer from memory and imbalance related issues. To address these issues, the spatial context of the segmentation consider by neural networks is typically restricted (e.g., by resolution reduction or analysis of environments of lower dimensions). Although efficient, such solutions hinder the ability of the neural networks to understand the complex 3D structures typical of the cerebrovascular system and to leverage this understanding for decision making.

**Approach:**

We propose a brain-vessels generative-adversarial-network (BV-GAN) segmentation model, that better considers connectivity and structural integrity, using prior based attention and adversarial learning techniques.

**Results:**

For evaluations, fivefold cross-validation experiments were performed on two datasets. BV-GAN demonstrates consistent improvement of up to 10% in vessel Dice score with each additive designed component to the baseline state-of-the-art models.

**Conclusions:**

Potentially, this automated 3D-approach could shorten analysis time, allow for quantitative characterization of vascular structures, and reduce the need to decrease resolution, overall improving diagnosis cerebrovascular vessel disorders.

## 1 Introduction

Stroke is the second leading cause of death as of 2016 and a major cause of disability worldwide.^1^ Detection of aneurysms is also of major clinical importance because hemorrhage can be fatal. An important method for detection and analysis of these pathologies is time-of-flight magnetic resonance angiography (TOF-MRA).^2^ The basic principle behind TOF-MRA imaging is flow-related-enhancement; stationary tissues in the imaged volume become magnetically saturated as a result of multiple repetitive radio frequency-saturation pulses. “Fresh” blood flowing into the imaged volume has not experienced these pulses and thus has a high initial magnetization, causing the signal from in-flowing blood to appear brighter than the background tissue.^3^ This imaging method is insensitive to in-plane flow and is susceptible to saturation effects, causing low sensitivity to slow flow vessels. Typically, analysis of the TOF-MRA relies on subjective visual interpretation by a neuroradiologist. Thus, accurate diagnosis is subjective, qualitative, and highly depends on the radiologist’s expertise. Common analysis methods include maximum intensity projections reconstruction of the three-dimensional (3D) scan. Such reconstruction techniques are typically combined with leveling, filtering, and surface display for proper visualization of the data. These projection-based processes reduce the acquired spatial resolution, increase sensitivity to noise, and vicariously may cause the concealment/deformation of small vasculature, damaging analysis sensitivity.^4^ Furthermore, angiogram outcome is highly biased by the projection axis, subjecting it to errors in the reconstruction of nonplanar vessels. Automatic segmentation methods of the cerebrovascular system based on TOF-MRA face significant challenges as blood vessels are complex and thin structures, constituting <5% of the entire scan volume and thus leading to a highly imbalanced task. This renders supervised learning methodologies harder as imbalanced data are known to cause significant bias and performance reduction.^5^

To address these drawbacks, we propose the brain-vessels generative-adversarial-network (BV-GAN) segmentation model that encourages connectivity and structural integrity using attention and adversarial learning techniques. First, we observe that blood vessels are highly nonplanar; hence segmenting them without sufficient volumetric context (e.g., on a single slice or in a 2.5D settings) is difficult (see Sec. 2 for more detail). For this reason, our model utilizes 3D TOF-MRA scan patches as input and produces a 3D cerebrovascular vessel segmentation map. To detect scarce vessel-containing voxels, addressing the aforementioned imbalance setting, we employ attention techniques. Our generator boosts attention to voxels more likely to contain vessels using latent space features extracted from a vessel segmentation map prior. Finally, a discriminator targets the preservation of vessel connectivity and structure because the statistics of vessel regions are likely to be similar across patches. This is one of the core advantages of our full 3D setting—the structure of a vessel is recognized and encouraged during training. This encouragement is optimized in multiscale, over the similarity between latent space features. Intuitively, such an approach aims to implicitly force preservation of long-short scale connections.

Through extensive evaluation on two datasets, we demonstrate how providing the model with multiscale attention feature maps and employing an adversarial training methodology boosts vessel precision and Dice-score and decreases the Hausdorff distance measure. These indicate improvements in vessel shape detection and integrity preservation. We further show that these concepts form the specially tailored system, which addresses the core challenges inherent to this task, yielding a satisfying improvement of 8% to 10% in vessel Dice score compared with the state-of-the-art (SOTA) 3D baselines (3D U-Net^6^ and Uception^7^). In addition, we also demonstrate how manual corrections of the ground truth (GT) annotations decrease data-driven errors, especially for our structure-aware approach. Following correction of about 30% of the data (which will be openly published), we show that this tedious slice level process yields a significant improvement in almost all measured metrics contributing to our system’s ability to target the cerebrovascular vessels (see Sec. 5.5). The BV-GAN model obtains a 0.76 Dice score with a 0.71 sensitivity, 0.86 precision, and 0.65 mm Hausdorff distance on the MIDAS dataset. In comparison, our baseline^6^ displays scores of 0.68, 0.73, 0.78, and 1.52, respectively. Furthermore, applying this automated segmentation technique gives neuroradiologists access to a nonaveraging-dependent 3D segmentation, thus enabling the analysis of vessels without resolution reduction. Such analysis could open the way for the detection of small vasculature and enable higher sensitivity-based segmentation. In addition, the 3D-based approach allows for the full TOF-MRA scan segmentation in nearly 20 s. This accurate and quick approach is a key first step in creating a fully automated system for the detection of various vascular malformations.

## 2 Related Work

Vessel segmentation poses a medical imaging challenge in various modalities and anatomical structures in general and in 3D TOF-MRA cerebrovascular vessels in particular. Early solutions include active contours, centerline-based, scale-space filtering, and statistical or hybrid models.^8^ These methods achieved notable results; however, some require assumptions regarding vessel gray-level distributions,^9^^,^^10^ which may differ between data sources. In addition, when facing large-scale dense 3D TOF-MRA data, computational time of these methods can extend up to several minutes per scan.^11^ Recent solutions incorporate supervised deep-learning (DL)-based models, leading to decreased runtime and improved segmentation results when compared with classical approaches. DL methods have been used in blood vessels segmentation for different human organs in various imaging modalities.^12^^–^^14^ An additional approach that targets computational efficiency and reduction in model parameters was proposed by Zhang et al.^15^ This work showed improved results on several 3D model benchmarks using a dilated model. This approach, however efficient, combined uniform dilation on a nonuniformly scattered network of vessels; hence it is incompatible with this problem. To exploit this nonuniform scatter of vessels, one may attempt to use 3D deformable convolutional networks (DCN)^16^ or spatial transformer-based sampling.^17^ These were experimented in the past with small input patches for retinal vessel segmentation^18^ and 3D multiorgan segmentation.^19^ These, however, differ greatly from our problem setup, including an extremely imbalanced larger search space. By contrast, our method efficiently traverses through the vessel search space by combining multiscale prior dependent latent features and an adversarial training methodology. These concepts yield superior performance when compared with Uception by Sanches et al. or vanilla 3D U-Net (see Sec. 5).

GANs have been used in the context of neuroimaging in the past for many applications, spanning from image reconstruction through disease progression models and brain decoding to the discussed goal of segmentation.^20^ However, most of these approaches do not tackle the 3D domain, probably due to the computational issues that it encumbers. In addition, GAN models are typically used for image-to-image translation (or cross-modality synthesis), image synthesis, and data augmentation. In concurrent work, Subramaniam et al.^21^ proposed a 3D GAN-based model for cerebrovascular segmentation. This work is orthogonal to ours as Subramaniam et al. proposed using GAN-based dataset augmentations (i.e., generating a large volume of examples using the GAN model, with self-generated supervision) to improve downstream training of an U-NET-based segmentation network. By contrast, we propose improving the U-NET-based segmentation itself using the GAN approach, which regardless could benefit from the additional data that Subramaniam et al. offer.

## 3 Materials

During this study the following two datasetes were used.

### 3.1 MIDAS Dataset

**Fig. 1:**
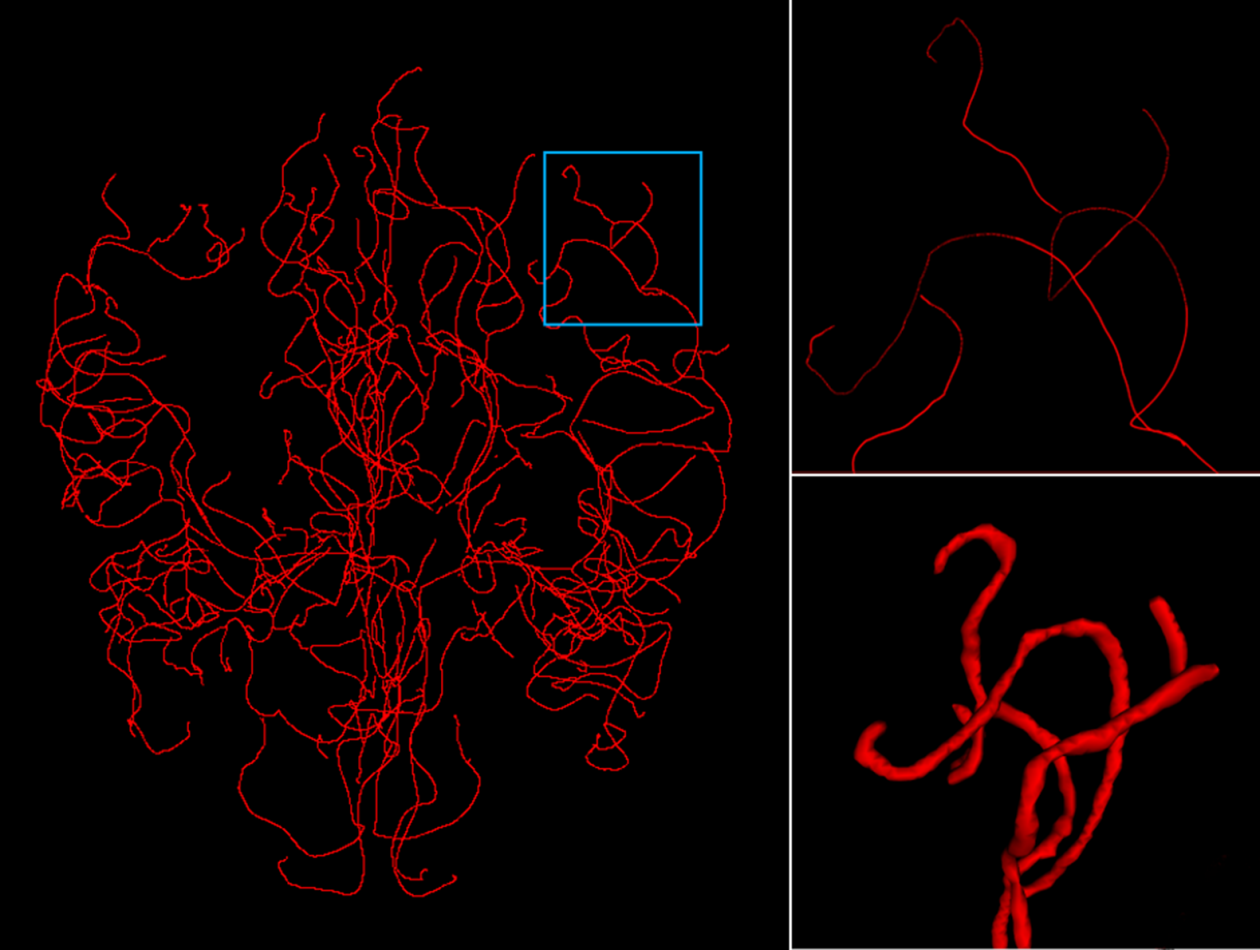
Input centerline data (left, ROI magnified in top right) are rendered into a tubular structure (bottom right) to generate the volumetric segmentation map.

The MIDAS public dataset^22^ is comprised of brain scans of 100 healthy subjects. Images were acquired on a 3T magnetic resonance imaging (MRI) under standardized protocols. In this paper, we use a subset of TOF-MRA scans acquired at a $0.5\times 0.5\times 0.5\;{\mathrm{mm}}^{3}$ resolution and shaped as 448×448×128  voxels in the “RAI” axis orientation. This subset of 42 scans also includes intracranial vasculature (centerline + radius) annotations, extracted from the TOF-MRA scans.^23^ The annotated scans were used for training, validation, and test sets in a fivefold cross-validation evaluation process. To render the 3D dense annotation maps, each centerline coordinate is modeled as a 3D isotropic sphere with the given radius (because no vessel directions were given). The primary maps then undergo smoothing and elliptic kernel erosion to achieve tubular morphology (see Fig. 1). These dense segmentation maps are used as ground-truth (GT). A subset of the MIDAS dataset annotations, containing 13 of the 42 maps, was manually corrected by junior raters and approved by an expert neuroradiologist (OA) from the Tel-Aviv Sourasky Medical Center (TASMC). The correction process targeted the removal of peripheral veins, accurate vessel edge segmentation, and connectivity improvement. Due to limited resources, this process was executed on only a subset. However, this initial investigation already demonstrates how sensitive this task is to noisy labels, hopefully motivating the future improvement of all annotated data. As is further discussed in Sec. 5.5, the correction process is even more meaningful when an adversarial loss is employed. This is attributed to the loss term’s explicit consideration of the local structure of the vessels, making discontinuities especially harmful.

### 3.2 Tel Aviv Sourasky Medical Center Dataset

This dataset is comprised of brain TOF-MRA scans of four healthy subjects. Scans were acquired at the TASMC on a 3T MRI under standardized TOF-MRA protocols. Scans were acquired at a $0.3125\times 0.3125\times 0.7\;{\mathrm{mm}}^{3}$ resolution and shaped as 624×768×200  voxels in oblique “RAI” orientation. Resampling was applied to match the 448×448×128  voxels shape of the MIDAS dataset to use an identically shaped prior, as mentioned in Sec. 4.1.2. Initial annotations were prepared using the pretrained segmentation model (see Sec. 5.2) and then manually corrected by two junior raters and revised by an expert neuroradiologist to produce GT vessel segmentation maps. The annotated scans are used as an additional test set, effectively examining generalization capabilities.

## 4 Methods

**Fig. 2:**
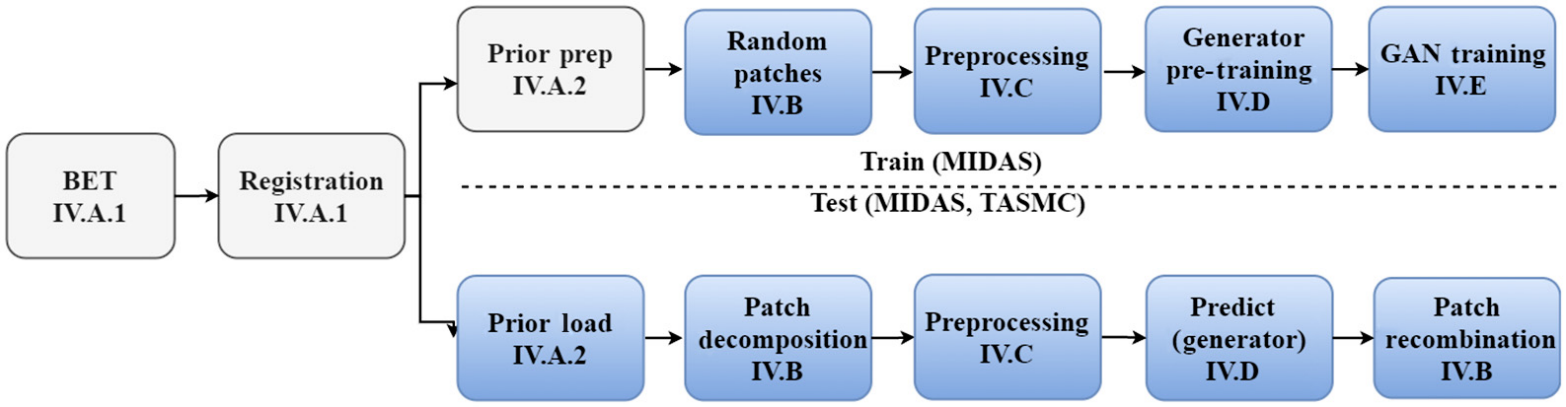
Proposed system diagram, offline/online steps depicted in gray/blue blocks, respectively. Each step index matches the relevant paper section.

We introduce an adversarial DL-based model for automatic cerebrovascular vessel segmentation from brain TOF-MRA scans. As shown in Fig. 2, our pipeline begins with an offline brain extraction (BET) and an affine registration step. Following these steps, during the training phase, we prepare the probabilistic vessel map anatomical prior. This is later leveraged as an attention map to mitigate the problems caused by the acutely imbalanced setting of the task. To properly segment cross-sectional vessels and preserve vessel connectivity, we introduce two additional concepts: (a) 3D patch-based training and (b) multiscale latent feature extraction. The 3D patch-based training balances memory limitations with spatial context. The intricate nonplanar structures of blood vessels imply that the network should consider the full 3D surrounding of a point to make an informed decision. Feeding 3D information, however, is very memory intensive, and hence lower resolution is typically used. Instead, we feed high resolution patches to the network, but they are of smaller spatial coverage. For global orientation, we rely on the prior map. The multiscale latent feature extraction is incorporated into the discriminator in an adversarial training methodology. This encourages structural similarity between the GT and predicted vessel latent features extracted by the segmentation model, encouraging the predicted vessel to be of plausible geometry, both in the large-scale general shape, and in the small-scale details. These principles target the preservation of long-short scale connections throughout the winding cerebrovascular vessels. At test time, we load the aforementioned vessel anatomical prior, align it to the target, and predict the segmentation map of the full scan on a per patch level. Using fivefold cross-validation and two separate source datasets (see Sec. 5), we demonstrate the robustness and generalization of our design to unseen data. These steps are surveyed in greater detail in the following sections.

### 4.1 Offline Steps and Prior Preparation

#### 4.1.1 BET and registration

The cerebrovascular vessels are scattered throughout the brain tissue. Hence, a brain extraction phase (BET) is necessary to remove nonrelevant tissues (bone, skin, fat, and air) and anatomy (neck, upper spinal cord, eyes, and mouth). The BET phase is performed in MATLAB R2019b using statistical parametric mapping (SPM)-based brain segmentation with the SPM12 tool.^24^ The BET process duration requires 3.5 min per scan on average. Following the BET phase, a broad-view single scan, aligned to the axis, is chosen from the MIDAS dataset (subject “Normal-071”), to which all scans and annotation maps are affinely registered; this is performed using SimpleElastix.^25^ The registration targets variability reduction in vessel locations, increasing coherence with the anatomical prior-based attention. The registration process takes 12.71 s per scan on average.

#### 4.1.2 Vessel anatomical prior preparation

**Fig. 3:**
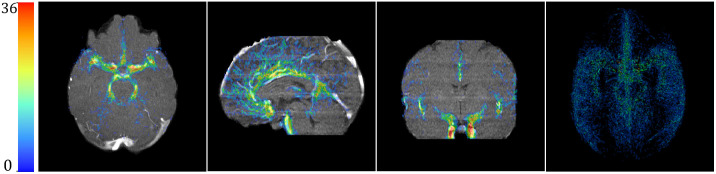
Attention prior heatmap displayed on MIDAS subject “Normal-071” from several viewpoints, from left to right: axial, sagittal, coronal views, and a 3D visualization of the whole map, from an axial point of view. The color bar depicts the number of times a voxel is occupied, from 0 to 36 (the training set size).

As mentioned, we harness a probabilistic vessel map anatomical prior-based guidance to provide our model with soft attention. The imbalanced setup of our vessel detection problem requires such instruction to limit the possible search-space and enable easier convergence to the scarce areas of interest throughout the scan. Such guidance should be given without compromising the uniform 3D structure and context, so it is vital to the task. The vessel anatomical prior is prepared at the beginning of each training fold out of the training set of 3D TOF-MRA vessel annotations. To create the prior, we sum for each voxel the number of times it is occupied in our training samples (36 patients in our case), according to the GT labels. To reduce noise, we remove (nullify) from the map voxels that are occupied by a blood vessel in <5% of the cases (i.e., in our case, we did not include any voxels that were occupied in only one patient). An example is shown in Fig. 3. The same prior is used throughout a specific training fold to perform the attention-based guidance (see Sec. 4.4). This includes both the validation process and during test time.

### 4.2 Random 3D-Patch-Based Training and Inference

**Fig. 4:**
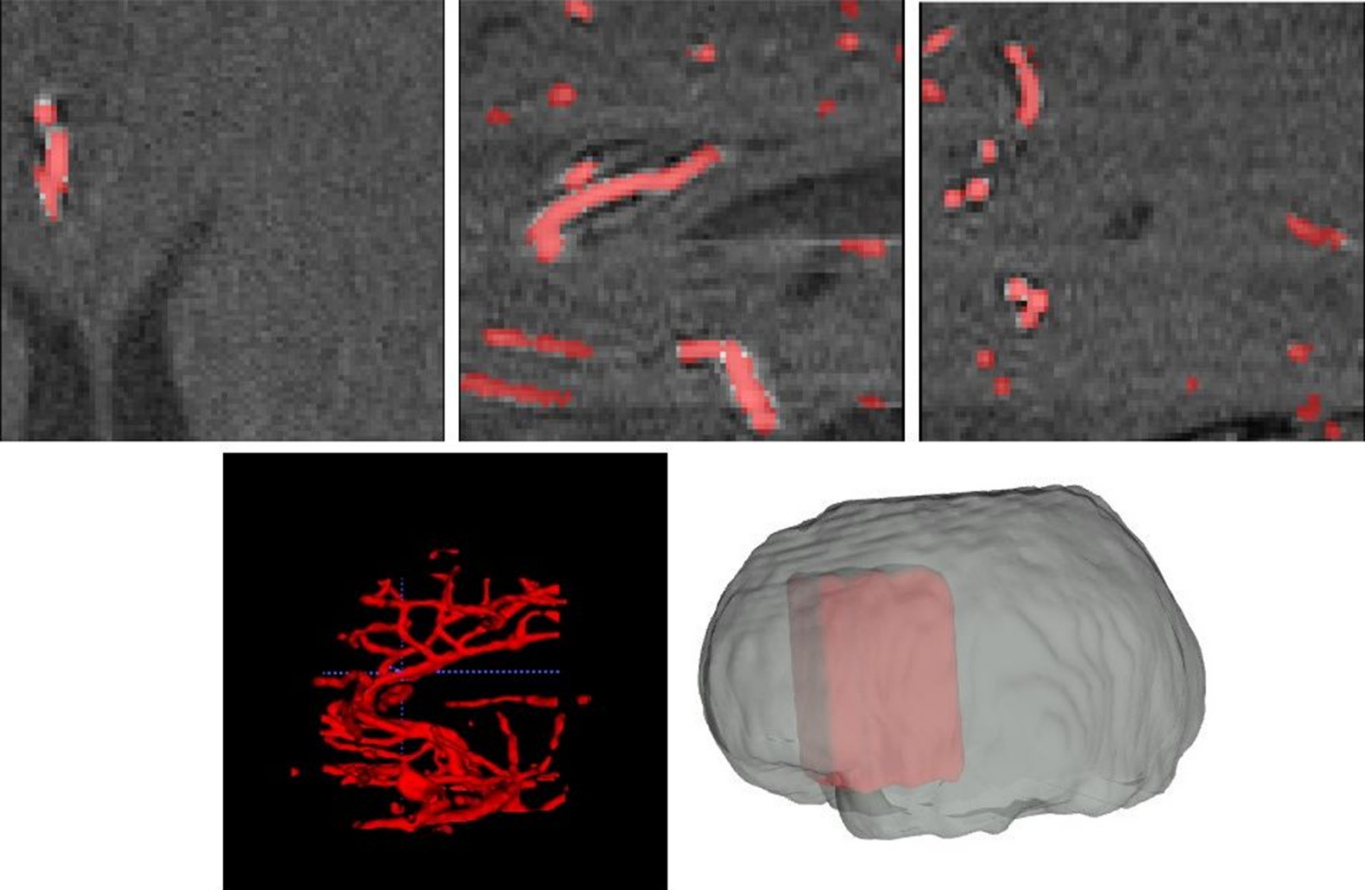
One patch (of 80×80×80  voxels) extracted from the brain. Top: axial (left), sagittal (middle), and coronal (right) cross-sections of a point in the patch. Bottom: 3D visualization of the vessels in the patch according to our segmentation (left) and the location of the patch within the brain (right).

Training data input scans are of 448×448×128 in volumetric resolution. Even if tightly bound around the actual brain area, this is an impractical size that exceeds memory limitations of prevalent GPUs. The straightforward solution would be to resize the scan such that it fits the used hardware at the expense of spatial resolution. However, the diameter of cerebrovascular vessels ranges from <1  mm up to a few mms at most.^26^ Therefore, it is crucial that the scan’s inner slice spacing and thickness is not be grossly damaged to preserve delicate high-resolution spatial information. Our proposed solution is to use random 3D-patch-based training. Using random 80×80×80 3D sampled patches from the brain’s tight bounding box, we ensure both the preservation of original scan resolution and the existence of anatomical context needed for the proper operation of our segmentation model. Examples of such patches are shown in Fig. 4. During test time, the whole brain cerebrovascular vessel segmentation is performed using a 3D sliding window of 80×80×80 patches with no overlap.

### 4.3 Preprocessing

The scan patch preprocessing step is composed from input data clipping and normalization to [0, 1]. Clipping is performed between [0,350] to remove noise. To enhance contrast, normalization is performed using linear rescaling between the 10th and 99th intensity percentile per scan, divided by the maximum intensity value. Both GT segmentation and prior maps are one-hot encoded into the 80×80×80×2 shape in which the first and second channels encode background and vessel maps, respectively. During training, both morphological (rotation, LR flips, and zoom with [0.9, 1.1] factor) and intensity (gamma noise) augmentations are applied, each with a different, empirically set probability. We did not notice high sensitivity of the system to these values. All morphological operations are applied identically on scan, GT, and prior data simultaneously to maintain voxel level consistency, whereas intensity augmentations are applied on scans alone.

### 4.4 Generator; Attention Prior-Based Segmentation

**Fig. 5:**
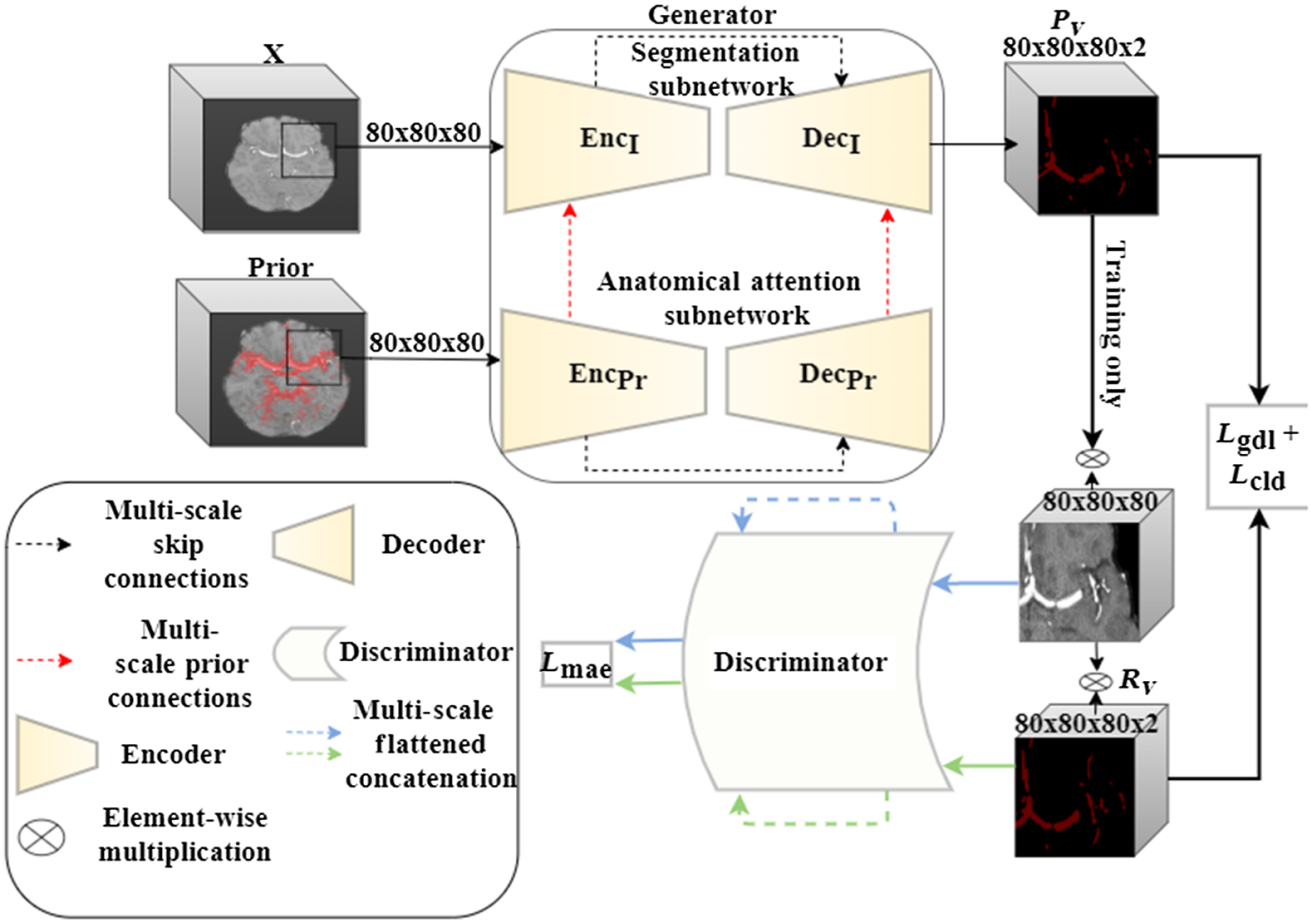
BV-GAN model architecture, surveyed thoroughly in Appendix 7.2. X is the scan patch input and $R_{v}$ and $P_{v}$ are the GT and predicted patch vessel masks, respectively.

The cerebrovascular vessels anatomy is distributed nonuniformly throughout the human brain. Such distribution requires a location-specific attention to highlight areas that are more likely to hold vessels. This form of distributed attention facilitates focusing computational resources on areas of higher relevance to the segmentation task at hand, without compromising the uniform 3D structure of the data. Therefore, we employ an anatomical attention subnetwork, i.e., a four-level 3D-Unet containing anatomical attention gates,^27^ in a multiscale fashion as described in Fig. 5. The prior features, which are extracted by a parallel 3D U-Net model, emphasize the importance of specific locations or areas in the scan features. This is done by applying a series of placewise additions/multiplications, channel concatenation, and joint 1×1×1 convolutions amplifying feature values in a location specific manner, as proposed by Sun et al.^27^ Attention gates were previously shown to incorporate anatomical prior during model training and to guide the segmentation process. Of course, other soft attention-based solutions are possible. These are, for example, as mentioned in Sec. 2, 3D DCN and spatial transformer-based sampling. However, we found that neither approach, when applied on large-scale 3D volumes for the scattered cerebrovascular vessels segmentation, obtains satisfactory results. These methods either create a significantly larger deformation field search space and cause model instability during training or maintain the regular grid-based sampling (see Appendix 7.1).

### 4.5 Discriminator; Multiscale Latent Feature Extractor

To enforce connectivity and to capture long- and short-range spatial relationships between voxels, we incorporate an additional hierarchical CNN model.^28^ This model targets the improvement of latent features similarity between masked GT scans and predicted vessel areas. This CNN serves as a discriminator in our adversarial training methodology as shown in Fig. 5. During training, the segmentation model generator aims to minimize both the segmentation loss and the adversarial loss, whereas the feature extractor discriminator aims to maximize the latter (loss terms are described in detail in the following section) in an alternating min-max counterplay. The feature extractor compares the latent features extracted from scan voxels proposed by the segmentation step with those in the GT that are annotated as containing vessels. Note that this configuration differs from feeding the entire scan patch and segmentation mask to the discriminator. The proposed configuration gives more emphasis to inspecting the visual distribution of vessel containing regions than to the segmentation structure. It turns out that this configuration demonstrated superior results, when compared with the former, providing spatial anatomical context in addition to the mask morphological structure. Being a learning aid for the segmentation model output, this part of the BV-GAN model is used only during training. Once convergence is achieved, the model performance is evaluated based on the output of the generator alone.

### 4.6 Loss Functions

The task of cerebrovascular vessel segmentation in TOF-MRA scans is highly imbalanced, as <5% of scan voxels contain blood vessels on average. To address this extreme imbalance and emphasize the importance of vessel containing voxels, a combination of weight dependent loss functions is used. During the segmentation model generator training, a loss function combined from the following two terms is employed:

1. Generalized Dice loss (GDL):^29^
  $$\ell_{\mathrm{gdl}}=1-2\frac{\overset{2}{\underset{l=1}{\sum }}w_{l}\overset{N}{\underset{n=1}{\sum }}R_{\ln}P_{\ln}}{\overset{2}{\underset{l=1}{\sum }}w_{l}\overset{N}{\underset{n=1}{\sum }}R_{\ln}+P_{\ln}},$$ (1)
  where R is the reference vessel segmentation, P is the predicted probabilistic vessel mask, l is the class index∈{background,vessel}, and n is the voxel index out of the total N scan voxels. $w_{l}$ is the weight coefficient calculated by $w_{l}=1/{\left(\overset{N}{\underset{n=1}{\sum }}R_{\ln}\right)}^{2}$. One can notice that $w_{l}$ is inversely proportional to the class prevalence, thus emphasizing uncommon labels (vessels) and underrating others (background).
2. Centerline Dice (CLD):^30^ a connectivity preserving metric seeking to maintain cerebrovascular vessels centerlines and bifurcations. The metric utilizes the vessel skeletons, calculated softly over predictions (and sharply over the GT).^30^ The skeletons $S_{R},S_{P}$ of reference and predicted vessel maps are extracted from $R_{v}$ and $P_{v}$, respectively. Using the skeletons, we calculate Topology precision and Topology sensitivity as
  $$T_{\mathrm{prec}}(S_{P_{v}},R_{v})=\frac{\left|S_{P_{v}}\cap R_{v}\right|}{\left|S_{P_{v}}\right|};\;T_{\mathrm{sens}}(S_{R_{v}},P_{v})=\frac{\left|S_{R_{v}}\cap P_{v}\right|}{\left|S_{R_{v}}\right|}.$$ (2)

To maximize both precision and sensitivity, Shit et al.^30^ constructed CLD as

$$\ell_{\mathrm{cld}}=2\frac{T_{\mathrm{prec}}(S_{P_{v}},R_{v})\times T_{\mathrm{sens}}(S_{R_{v}},P_{v})}{T_{\mathrm{prec}}(S_{P_{v}},R_{v})+T_{\mathrm{sens}}(S_{R_{v}},P_{v})}.$$ (3)

Both terms have an equal contribution to the combined additive loss term used to train the soft attention prior-based segmentation model.

3. Feature loss: As discussed in Sec. 4.5, we employ a multiscale feature loss between the predicted and GT vessel containing adjacent areas at multiple layers of the discriminator model. The adversarial model loss component is defined as
  $$\underset{\theta_{G}}{\min}\;\underset{\theta_{D}}{\max}\;\ell_{\mathrm{mae}}=\frac{1}{L}\overset{L}{\underset{i=1}{\sum }}{\left\|f_{D}^{i}(x\circ P_{v})-f_{D}^{i}(x\circ R_{v})\right\|}_{1},$$ (4)
  where L is the total number of layers (i.e., scales) in the discriminator network (five in the case of our architecture), x is a single input scan patch in the current batch, and $f_{D}^{i}$ represents the hierarchical features extracted from image patch x in layer i by the discriminator model.

While training the soft attention prior-based segmentation model and discriminator network simultaneously, the following combined loss function is employed:

$$\ell_{\mathrm{BV}\text{-}\mathrm{GAN}}=\ell_{\mathrm{gdl}}+\ell_{\mathrm{cld}}+\ell_{\mathrm{mae}}.$$ (5)

It is of note that all loss functions are averaged over the batch size.

### 4.7 Implementation Details

The generator’s segmentation task of predicting vessel locations is considerably more complex than that of the discriminator for distinguishing between GT and prediction-based latent features; therefore, the following steps are taken:

(a) The segmentation model is pretrained separately for up to 100 epochs and stopped early preconvergence.
(b) During adversarial training, the discriminator is iterated every three generator iterations.

Training (validation) data is iterated twice at the end of each epoch with different random crops to stabilize average loss and metrics due to the small number of available MIDAS dataset scans. During both segmentation pretraining and adversarial training, the vessel Dice coefficient ($V_{\mathrm{DSC}}$) is monitored, and the learning rate decay is used during metric plateau. Adversarial training is stopped early after up to 150 epochs, after which the monitored metric shows no significant improvement. The initial learning rate during segmentation model pretraining is 5e–3, and the decayed learning rate at the end of this step is used for generator initialization in the adversarial step. The discriminator’s initial learning rate is 1e–4. A batch size of six patches is used. Adam optimizer is used for all models with 0.9 momentum. All models are trained with three Nvidia GeForce RTX 2080 Ti GPUs. The environment is built in Pyhton 3.6 using the Keras library.

## 5 Experiments and Results

In all subsequent experiments, results were obtained using a fivefold subjects cross-validation to ensure that results are indifferent to data division and are of low variability. In each fold, MIDAS dataset scans were divided into train (36), validation (3), and test (3). The metrics used to evaluate vessel segmentation performance during training/validation/test are vessel sensitivity, precision and Dice score, as follows:

$$V_{\mathrm{Sens}}=\frac{\mathrm{TP}}{P}=\frac{R_{v}\cap P_{v}}{R_{v}}V_{\mathrm{DSC}}=2\frac{\mathrm{TP}}{2\mathrm{TP}+\mathrm{FP}+\mathrm{FN}}=2\frac{R_{v}\cap P_{v}}{R_{v}\cup P_{v}}\;V_{\mathrm{Prec}}=\frac{\mathrm{TP}}{\mathrm{TP}+\mathrm{FP}}=\frac{R_{v}\cap P_{v}}{R_{v}\cap P_{v}+{\overset{¯}{R}}_{v}\cap P_{v}},$$ (6)

where TP, FP, and FN are the vessel segmentation true-positives, false-positives, and false-negatives, respectively. The validation set $V_{\mathrm{DSC}}$ score averaged across 3D patch batches is monitored in the end of each training epoch. Once convergence (determined when reaching a $V_{\mathrm{DSC}}$ plateau) is achieved, training is terminated, and the above metrics are calculated and averaged on full size test scans as shown in Sec. 4.2. In addition, we employ another metric—vessel maps Hausdorff distance:

$$V_{D_{\mathrm{Haus}}}=\max\{\underset{x\in R_{v}}{\sup}\;\underset{y\in P_{v}}{\inf}\;d(x,y),\underset{y\in P_{v}}{\sup}\;\underset{x\in R_{v}}{\inf}\;d(x,y)\},$$ (7)

where d(x,y) is the Euclidean distance between voxels x and y. Test metrics are also evaluated separately on the TASMC dataset described in Sec. 3.2. The following five depicted experiments target the contribution evaluation of each of the BV-GAN model design choices: starting with the evaluation of 3D SOTA models as baseline and followed by the introduction of the soft attention and adversarial training concepts.

### 5.1 Baseline 3D U-Net

In this experiment, a vanilla 3D U-Net is used, identical to the segmentation model described in Fig. 5 and excluding the anatomical prior network and gates. Model hyperparameters are optimized using a combined $\ell_{\mathrm{gdl}}+\ell_{\mathrm{cld}}$ loss function, and the test sets performance evaluation is displayed in row 1 in Table 1. Similar results are obtained when implementing Uception (Table row 2), designed by Sanches et al.^7^^,^^31^ These experiments validate our claim that vanilla 3D model performance is limited, and significant improvement warrants model guidance during training. As is seen, these models do not generalize well on unseen data, such as TASMC TOF-MRA scans.

**Table 1:** Fivefold cross-validation experiment results. Each row depicts the folds mean and standard deviation of an individual experimented model over both datasets.

#	Model	MIDAS				TASMC			
		$V_{\mathrm{DSC}}$	$V_{\mathrm{Sens}}$	$V_{\mathrm{Prec}}$	$V_{\mathrm{Haus}}$	$V_{\mathrm{DSC}}$	$V_{\mathrm{Sens}}$	$V_{\mathrm{Prec}}$	$V_{\mathrm{Haus}}$
1	3D U-Net	0.68 ± 0.03	**0.73 ± 0.01**	0.78 ± 0.03	1.52 ± 0.16	0.58 ± 0.04	0.55 ± 0.04	0.69 ± 0.05	1.32 ± 0.27
2	Uception	0.66 ± 0.02	0.71 ± 0.02	0.81 ± 0.01	1.42 ± 0.19	0.61 ± 0.01	0.55 ± 0.02	0.74 ± 0.02	1.31 ± 0.33
3	3D U-Net + Prior	0.72 ± 0.02	0.71 ± 0.03	0.82 ± 0.04	0.99 ± 0.24	0.64 ± 0.01	0.56 ± 0.03	0.77 ± 0.01	1.22 ± 0.30
4	BV-GAN	**0.76 ± 0.03**	0.71 ± 0.05	**0.85 ± 0.01**	**0.65 ± 0.11**	**0.70 ± 0.01**	**0.68 ± 0.05**	**0.79 ± 0.06**	**0.80 ± 0.06**
5	BV-GAN W/O Prior	0.72 ± 0.03	0.67 ± 0.04	0.80 ± 0.05	1.38 ± 0.33	0.59 ± 0.03	0.56 ± 0.04	0.75 ± 0.02	1.23 ± 0.43
6	BV-GAN (GDL)	0.72 ± 0.01	0.70 ± 0.02	0.80 ± 0.03	1.14 ± 0.20	0.62 ± 0.01	0.56 ± 0.03	0.71 ± 0.02	1.25 ± 0.23
7	BV-GAN (CLD loss)	0.71 ± 0.03	0.71 ± 0.04	0.81 ± 0.03	1.15 ± 0.21	0.62 ± 0.02	0.55 ± 0.02	0.73 ± 0.03	1.20 ± 0.21
8	BV-GAN (W/O MC)	0.71 ± 0.02	0.71 ± 0.03	0.81 ± 0.03	1.12 ± 0.21	0.62 ± 0.03	0.54 ± 0.02	0.73 ± 0.03	1.25 ± 0.10
9	3D U-Net (W/O MC)	0.66 ± 0.01	0.70 ± 0.03	0.75 ± 0.04	1.61 ± 0.57	0.55 ± 0.07	0.55 ± 0.06	0.71 ± 0.07	1.79 ± 0.33

Note: Bold characters in each column represents the top performing model (rows) according to each metric (column) as calculated on each of the two datasets (MIDAS/TASMC) results.

### 5.2 Attention Gated 3D U-NET

As stated in Sec. 4.4, attention-based guidance aides model learning, leading to performance improvement. To demonstrate this claim, we perform two experiments using the following models:

(a) 3D U-Net + prior: a segmentation model combining the baseline 3D U-Net and the prior attention model. The model is trained using the same combined loss terms described in Sec. 5.1.
(b) BV-GAN W/O (without) prior: the model received by ablating the prior 3D U-Net model and all anatomical gates from the architecture described in Fig. 5. This is trained using the same combined loss described (Sec. 5).

**Fig. 6:**
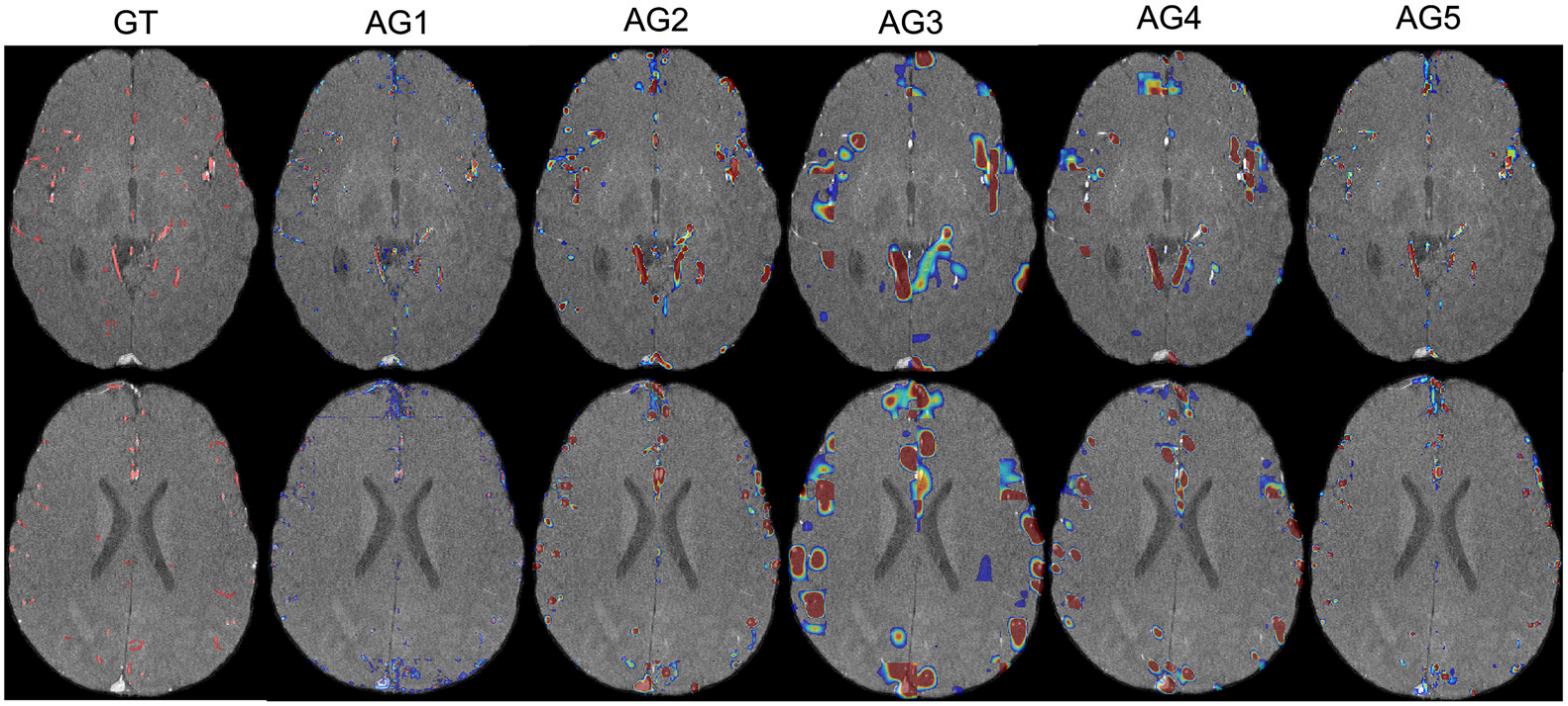
GT segmentation masks and anatomical AG (attention gates) 1 to 5 weighted sum of feature maps (columns) displayed as heatmaps on two different subject input slices (rows).

The performance evaluation of models (a) and (b) is displayed in Table 1 rows 3 and 5, respectively. Improvement is observed in almost all performance metrics, compared with 3D U-Net baseline (Table row 1). Furthermore, a decrease in said measurements is detected in the ablated model compared with the full BV-GAN model (Table row 4). Both trends indicate the added value in using the vessel map prior. Further justification to our claim regarding the amplification of significance of vessel containing voxels using the anatomical gates is shown in Fig. 6. This demonstrates the high weight values provided in vessel adjacent areas in the anatomical gates.

### 5.3 BV-GAN

This experiment incorporates all components described in Fig. 5. As depicted in Sec. 4.7, the training is composed of the following steps:

1. Pretraining of the soft attention gated 3D U-Net for 100 epochs and early stopping preconvergence. This is done to both warm start the generator and to avoid local minima often found by the optimizer that damage training initialization. The pretraining phase targets the minimization of the same combined loss described in Sec. 5.1.
2. Joint model adversarial training, iterating between soft attention gated 3D U-Net (generator) and multiscale-based latent feature extractor (discriminator) for 150 epochs, while minimizing the loss function in Eq. (5).

**Fig. 7:**
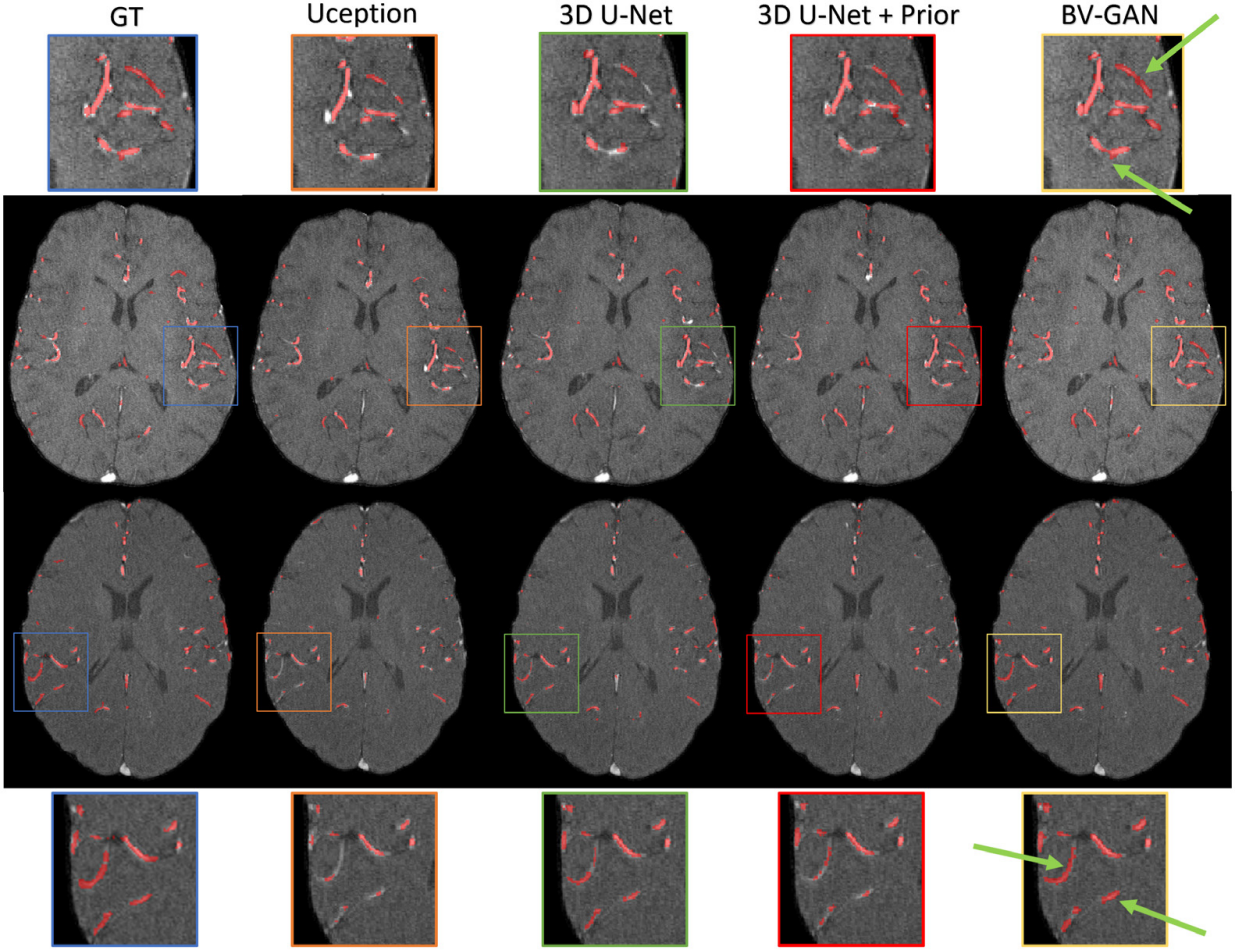
Qualitative comparison of segmentation outputs for two slices (rows) and various approaches (columns). Zoomed-in areas highlight major differences. Note how our method prefers the connected components (green arrows).

As shown in Table 1 row 4, BV-GAN demonstrated superiority over all other models in almost all metrics, specifically when compared with the 3D U-Net + prior, emphasizing the adversarial training contribution during model training to the final segmentation model performance. A qualitative comparison of the adversarial training contribution to the final performance is shown in Fig. 7, demonstrating improvement in vessel connectivity and integrity and fewer missed vessels.

### 5.4 Loss Functions Ablation Study

To determine the added value of using our $\ell_{\mathrm{gdl}},\ell_{\mathrm{cld}}$ combined segmentation loss function, we ablated each of the separate function components. Rows 6 and 7 of Table 1 contain the single (gdl/cld) loss function training results. These demonstrate a deterioration in all measured parameters on both datasets, indicating that the combination of the imbalance weighting gdl term and vessel morphology preservation cld loss synergize well. The sensitivity drop on the unseen TASMC data indicates that vessel segmentation generalization is highly dependent on both loss components. In addition, a Hausdorff distance increase was measured when each of the loss functions was ablated. Such trends provide further support for the increasing difference in vessel morphology between GT and the predicted vessel segmentation maps.

### 5.5 Corrected Subset Effect

During the MIDAS dataset dense segmentation maps preparation process, we noticed that the segmentation maps contain several error types: (a) dilated/eroded vessel segmentation—resulting from the sphere shape-based estimation, contributing either FN or FP vessel voxels accordingly. (b) Nonconnected vessels and false connections between adjacent vessels—also resulting from the above-mentioned rendering technique. (c) Undesired veins—the original centerline annotations include some veins, mostly in the periphery, as part of the cerebrovascular arteries annotation. Our MC (manual-correction) process tackled the above errors on a 30% subsample of the MIDAS dataset. When comparing between Table 1 rows 8 and 4, we notice that the correction process induces an improvement in most measured metrics. This indicates the dependency on accurate and reliable training data. We further notice that our method boosts performance more substantially when operating on the semicorrected data (comparing improvement between rows 1→4 and 9→8). This result implies that our models indeed leverage the geometry typical to the annotated data and hence the gains from reconnected vessels and other defect corrections more than a method that is less context-aware.

## 6 Discussion and Conclusion

Our research targets the accurate and efficient segmentation of the cerebrovascular vessels from 3D brain TOF-MRA scans. Inspired by soft attention-based DL and GANs, we proposed our BV-GAN model. Designed to handle the extremely imbalanced nature of the brain vessels segmentation problem, our model utilizes both guidance and multiscale long-short spatial context. Through extensive cross-validation on two separate datasets, we have examined the contribution of each of our design choices. The use of a 3D patch-based method enabled the full scan segmentation in about 20 s (12.71 s for registration and 8.17 s for all other pipeline steps combined), excluding the offline BET process (3.5 min). This approach saved run-time (in comparison with a slice-based sliding window approach), alleviated the need for scan resolution reduction, and assisted cross-sectional vessel segmentation with spatial context. The affine-registered input enabled the use of the vessel prior map. When used, such a map provides soft attention to voxels that are in highly occupied regions, boosting performance on both datasets. Our loss function weighs the scarce vessel-containing voxels heavily while aiming for structural integrity. The incorporation of an adversarial training methodology requiring multilevel latent space similarity provides additional supervision and generalization capabilities. In addition, we have produced openly accessible manually corrected scans through a tedious slice level inspection by both a junior rater and an expert neuroradiologist. As can be seen in our reported experiments, the corrected data annotations boost the performance of our GAN-based system much more than for the baseline counterparts. This nicely demonstrates how our discriminators learn the local behavior of a typical blood vessel. To further improve the segmentation performance for cases in which vessels are enlarged or in atypical locations (e.g., Moya-Moya), better priors should be harnessed. These include age- or pathology-grouped vessel priors, with corresponding additional training data.

Our proposed segmentation approach is a first step in developing an automated vessel pathology detection system, providing quantitative measurements for such pathological conditions. In the future, this tool could be extended to also provide accurate vessel width and specific vessel identification and eventually to develop a better tool for identifying different pathologies and diseases.

## 7 Appendix

### 7.1 Appendix A

**Fig. 8:**
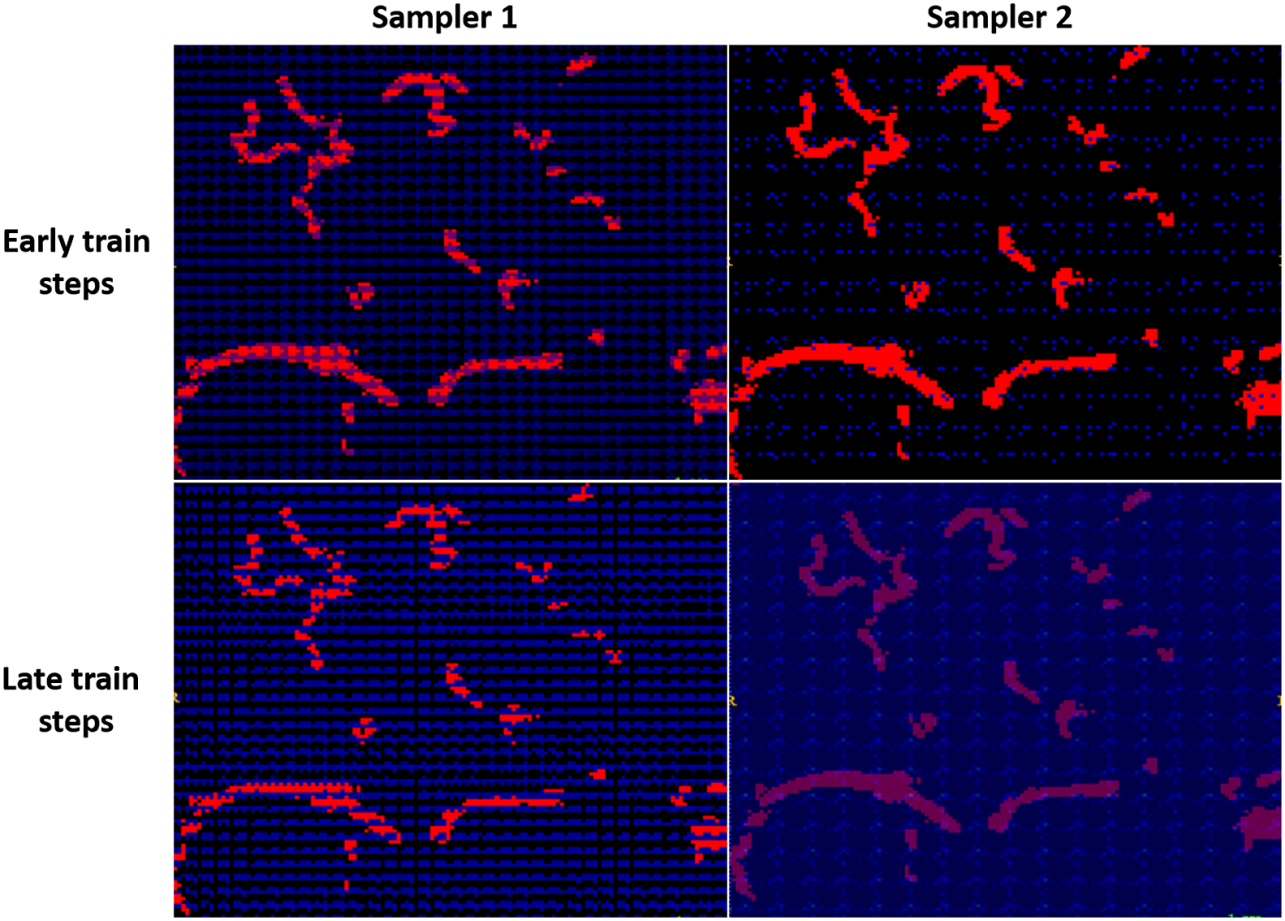
OBELISK-Net model samplers 1 and 2 (columns) sampling frequencies displayed as a heatmap on vessel GT masks, as received in early and late steps during model training (rows). Blue color indicates the low sampling frequency.

During our exploration of possible approaches to alleviating the DL model’s search for the scarce vessel voxels and limit the search space, two of the main ideas were to use spatial transformers and deformable convolution layers. The two concepts aim to incorporate layers into the model in which both filter coefficients and spatial filter offsets (from the uniform sampling method) are learned during training to target the high relevance areas in the input. Hence, we attempted to employ the intuitive notion of letting these learned deformations give emphasis to the interesting regions in the input, in light of the great spatial imbalance in the data. The spatial transformers were tested using OBELISK-Net.^19^ While training the OBELISK-Net model on our cerebrovascular vessel segmentation task using the same size patches as input, we visualized the sampling frequencies of each patch voxel as received from the model’s two existing samplers (spatial transformers), both in primary and advanced time points during model training. This was done to identify whether the sampling frequency of vessels containing voxels increases during training. As shown in Fig. 8, this convergence did not occur, and a uniform grid sampling persistently emerged from the training.

In our attempt to integrate deformable convolution^16^ layers into our basic 3D U-Net model, we exchanged the regular 3D convolution layers with their deformable counterparts. Our initial experiment included the exchange of all convolution layers in the first encoder–decoder scale (operating on the original shape volume), which we denote as deformable scale 0 3D U-Net (DS 0 3D U-Net). In this experiment, we increased the permitted offset from the regular grid-based sampling up to an offset of 40 voxels in each axis (a half of the input size). Results are displayed in Table 2. They demonstrate that, as the search space limitation is lifted (i.e., offset is larger), performance decreases, indicating that learning large 3D offsets causes model performance instability.

**Table 2:** Best validation set vessel Dice obtained during training of DS 0 3D U-Net. The maximal deformable layers offset (in voxels) is increased throughout experiments.

N	Maximal offset	Validation set vessel Dice
1	5	0.67
2	10	0.60
3	15	0.53
4	20	0.51
5	25	0.48
6	30	0.45
7	35	0.38
8	40	0.23

### 7.2 Appendix B

**Fig. 9:**
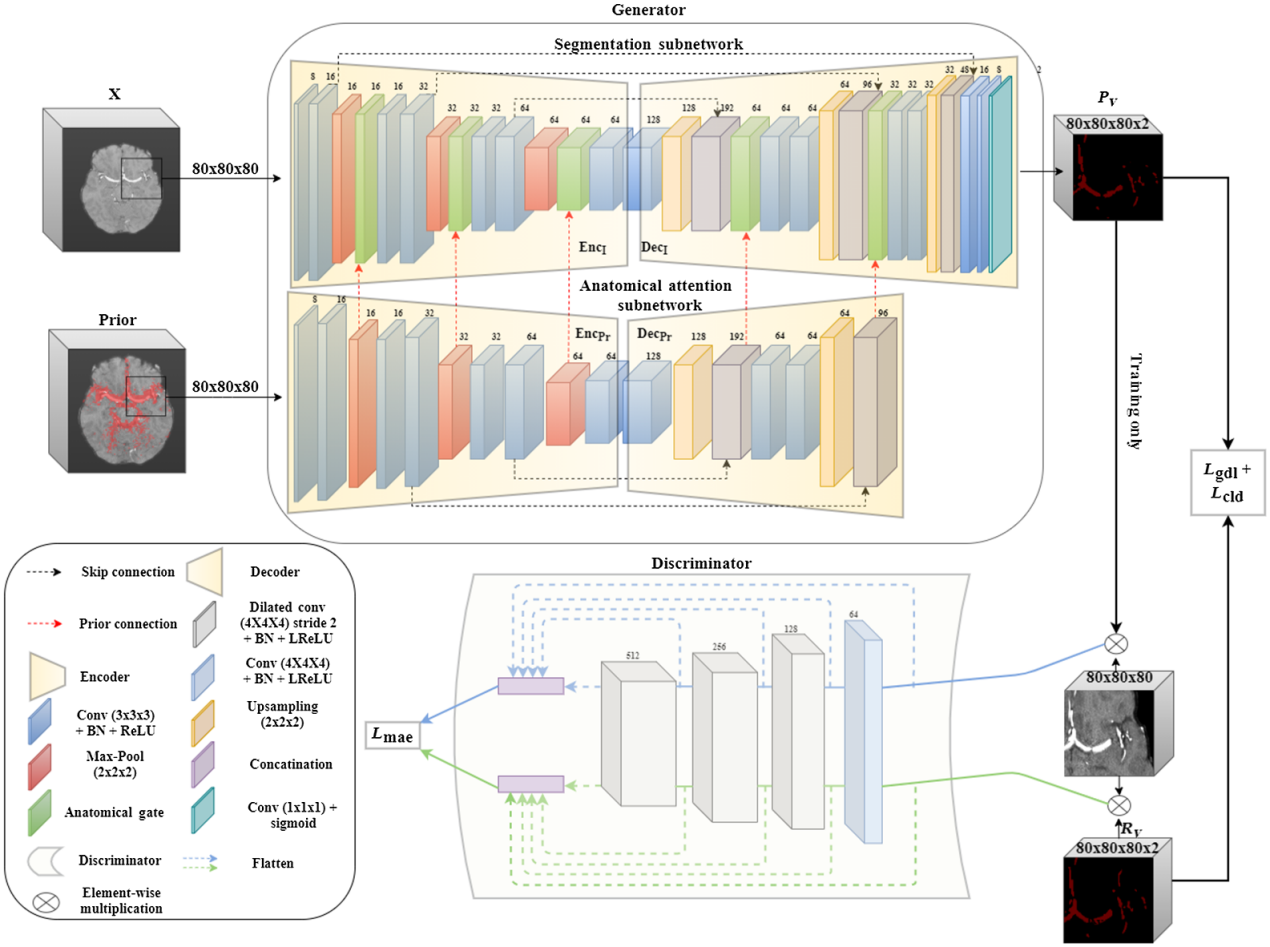
BV-GAN full model architecture. Depicting the 3D model scan and corresponding prior patches as inputs to both the generator’s segmentation and anatomical attention subnetworks accordingly. Followed by the multiscale discriminator used during adversarial training. Loss functions are specified in rectangles.

**Fig. 10:**
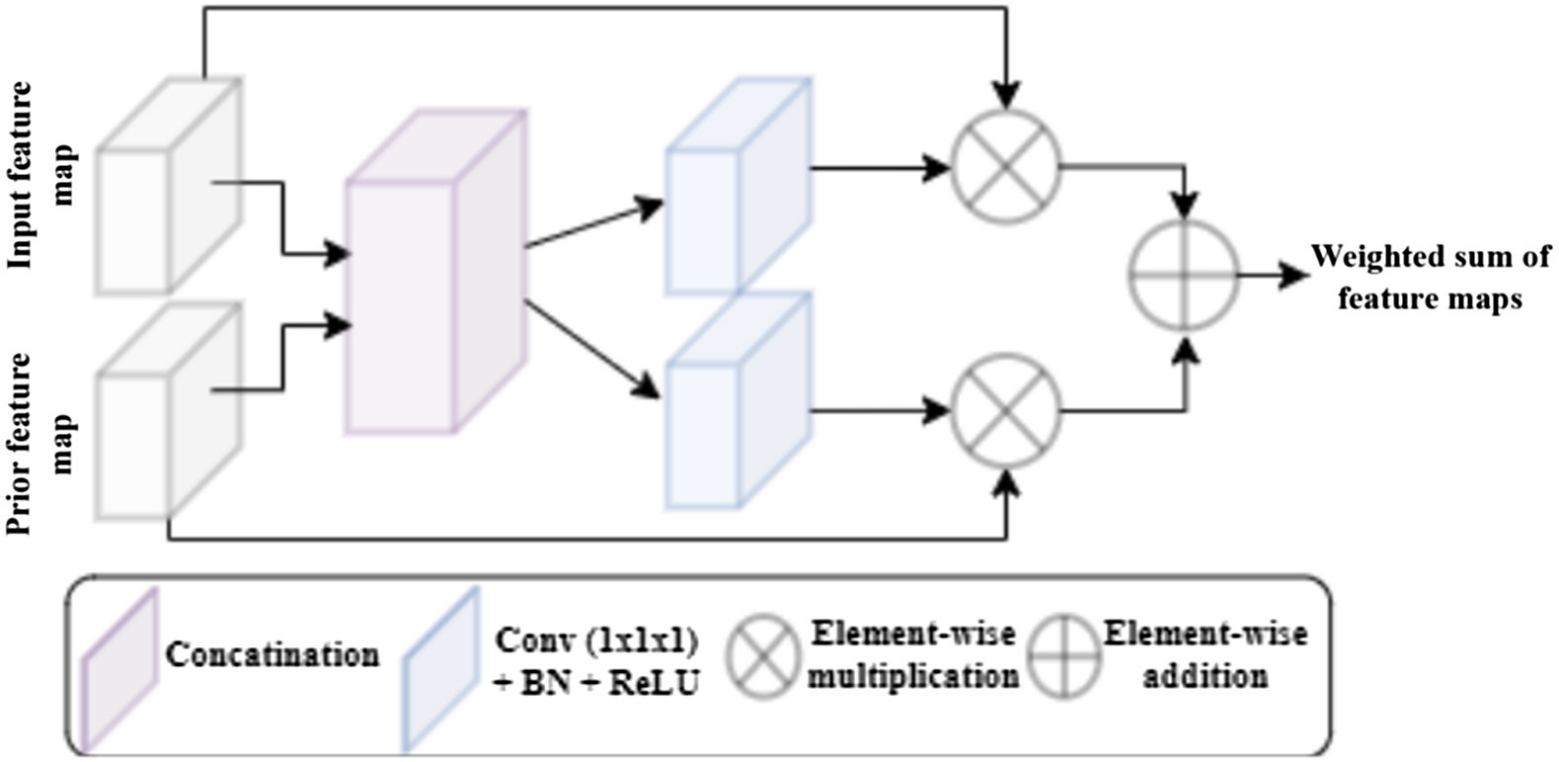
Anatomical gates architecture.

The full BV-GAN model architecture, displaying all incorporated subnetworks with compositing layers is depicted in detail in Fig. 9. The BV-GAN model anatomical gates enable the integration of multiscale anatomical prior-based soft attention into the segmentation network through a series of layers, depicted in detail in Fig. 10. Each gate produces a weighted sum of both prior and scan extracted features through a set of separate and joint convolution, multiplication, and addition operations.

## References

[1] World Health Organization [WHO], “The top 10 causes of death,” 2018, https://www.who.int/news-room/fact-sheets/detail/the-top-10-causes-of-death.

[2] Radwan M. E. Aboshaera K. O., “Magnetic resonance angiography in evaluation of acute intracranial steno-occlusive arterial disease,” Egypt. J. Radiol. Nucl. Med. 47(3), 903–908 (2016). 10.1016/j.ejrnm.2016.05.023

[3] Saloner D., “The AAPM/RSNA physics tutorial for residents. An introduction to MR angiography,” Radiographics 15(2), 453–465 (1995). 10.1148/radiographics.15.2.7761648 7761648

[4] Anderson C. M. et al., “Artifacts in maximum-intensity-projection display of MR angiograms,” Am. J. Roentgenol. 154(3), 623–629 (1990). AJROAM 0092-5381 10.2214/ajr.154.3.2106232 2106232

[5] Buda M. Maki A. Mazurowski M. A., “A systematic study of the class imbalance problem in convolutional neural networks,” Neural Networks 106, 249–259 (2018). NNETEB 0893-6080 10.1016/j.neunet.2018.07.011 30092410

[6] Cicek O. et al., “3D U-Net: learning dense volumetric segmentation from sparse annotation,” in Proc. MICCAI, Athens, pp. 424–432 (2016).

[7] Sanches P., “Uception-3D medical image segmentation with CNN,” 2018, https://github.com/SANCHES-Pedro/Segmentation_3D_DeepLearning.

[8] Taher F. et al., “A review on the cerebrovascular segmentation methods,” in Proc. ISSPIT, Louisville, Kentucky, pp. 359–364 (2018).

[9] Gao X. et al., “A fast and fully automatic method for cerebrovascular segmentation on time-of-flight (TOF) MRA image,” J. Digital Imaging 24(4), 609–625 (2011). JDIMEW 10.1007/s10278-010-9326-1 PMC3138936 20824304

[10] Wen L. et al., “A novel statistical cerebrovascular segmentation algorithm with particle swarm optimization,” Neurocomputing 148(19), 569–577 (2015). NRCGEO 0925-2312 10.1016/j.neucom.2014.07.006

[11] Tian Y. Liu Z. Zhao S., “Vascular segmentation of neuroimages based on a prior shape and local statistics,” Front. Inf. Technol. Electron. Eng. 20(8), 1099–1108 (2019). 10.1631/FITEE.1800129

[12] Liskowski P. Krawiec K., “Segmenting retinal blood vessels with deep neural networks,” IEEE Trans. Med. Imaging 35(11), 2369–2380 (2016). ITMID4 0278-0062 10.1109/TMI.2016.2546227 27046869

[r13] Kitrungrotsakul T. et al., “VesselNet: a deep convolutional neural network with multi pathways for robust hepatic vessel segmentation,” Comput. Med. Imaging Graphics 75, 74–83 (2019). 10.1016/j.compmedimag.2019.05.002 31220699

[14] Meijs M. et al., “Cerebral artery and vein segmentation in four-dimensional CT angiography using convolutional neural networks,” Radiol.: Artif. Intell. 2(4), 74–83 (2020). 10.1148/ryai.2020190178 PMC8082400 33937832

[15] Zhang Y. Chen L., “DDNet: a novel network for cerebral artery segmentation from MRA IMAGES,” in Proc. 12th CISP-BMEI, Huaqiao, Suzhou, pp. 1–5 (2019).

[16] Dai J. et al., “Deformable convolutional networks,” in Proc. ICCV, Venice, pp. 764–773 (2017).

[17] Jaderberg M. Simonyan K. Zisserman A., “Spatial transformer networks,” in Proc. 28th NeurlPS, Montreal, pp. 2017–2025 (2015).

[18] Jin Q. et al., “DUNet: a deformable network for retinal vessel segmentation,” Knowl.-Based Syst. 178(15), 149–162 (2019). KNSYET 0950-7051 10.1016/j.knosys.2019.04.025

[19] Heinrich M. Oktay O. Bouteldja N., “OBELISK-Net: fewer layers to solve 3D multi-organ segmentation with sparse deformable convolutions,” Med. Image Anal. 54, 1–9 (2019). 10.1016/j.media.2019.02.006 30807894

[20] Laino M. E. et al., “Generative adversarial networks in brain imaging: a narrative review,” J. Imaging 8, 83 (2022). 10.3390/jimaging8040083 35448210 PMC9028488

[21] Subramaniam P. et al., “Generating 3D TOF-MRA volumes and segmentation labels using generative adversarial networks,” Med. Image Anal. 78, 102396 (2022). 10.1016/j.media.2022.102396 35231850

[22] Bullitt E. et al., “Vessel tortuosity and brain tumor malignancy: a blinded study,” Acad. Radiol. 12, 1232–1240 (2005). 10.1016/j.acra.2005.05.027 16179200 PMC2517122

[23] Tube T. K., “Segmentation, registration and analysis of tubular structures in images,” 2018, https://public.kitware.com/Wiki/TubeTK/Data.

[24] Friston K. et al., “SPM12,” 2020, https://www.fil.ion.ucl.ac.uk/spm/software/spm12/.

[25] Marstal K. et al., “SimpleElastix: a user-friendly, multi-lingual library for medical image registration,” in Proc. WBIR, Las Vegas, Nevada, pp. 134–142 (2016).

[26] Mujagic S. et al., “The inner diameter of arteries of the circle of Willis regarding gender and age on MRA,” Acta Med. Saliniana 42(2), 6–12 (2013). 10.5457/342

[27] Sun L. et al., “Anatomical attention guided deep networks for ROI segmentation of brain MR images,” IEEE-TMI 39(6), 2000–2012 (2019). 10.1109/TMI.2019.2962792 31899417

[28] Xue Y. et al., “SegAN: adversarial network with multi-scale L1 loss for medical image segmentation,” Neuroinformatics 16(3), 383–392 (2018).1539-2791 10.1007/s12021-018-9377-x 29725916 PMC13344194

[29] Sudre C. et al., “Generalised dice overlap as a deep learning loss function for highly unbalanced segmentations,” in Proc. MICCAI Workshop on DLMIA, Quebec City, Quebec, pp. 240–248 (2017). 10.1007/978-3-319-67558-9_28 PMC7610921 34104926

[30] Shit S. et al., “clDice: a topology-preserving loss function for tubular structure segmentation,” in Proc. IEEE/CVF Conf. Comput. Vision and Pattern Recognit., pp. 16560–16569 (2020).

[31] Sanches P. et al., “Cerebrovascular network segmentation on MRA images with deep learning,” in Proc. IEEE 16th ISBI, Venice, pp. 768–771 (2019).

